# Spectroscopic-Chemical Fingerprint and Biostimulant Activity of a Protein-Based Product in Solid Form

**DOI:** 10.3390/molecules23051031

**Published:** 2018-04-27

**Authors:** Andrea Ertani, Ornella Francioso, Erika Ferrari, Michela Schiavon, Serenella Nardi

**Affiliations:** 1 Dipartimento di Agronomia, Animali, Alimenti, Risorse Naturali e Ambiente (DAFNAE), Università di Padova, Viale dell’Università 16, 35020 Legnaro (Padova), Italy; michela.schiavon@unipd.it (M.S.); serenella.nardi@unipd.it (S.N.); 2 Dipartimento di Scienze e Tecnologie Agro-Alimentari (DISTAL), Università di Bologna, Viale Fanin 40, 40127 Bologna, Italy; ornella.francioso@unibo.it; 3 Dipartimento di Scienze Chimiche e Geologiche, Università di Modena e Reggio Emilia, via Campi, 103-41125 Modena, Italy; erika.ferrari@unimore.it

**Keywords:** antioxidants, FTIR, ^13^C CPMAS NMR, HR MAS NMR

## Abstract

A solid biostimulant (AA309) obtained through thermobaric hydrolysis applied on trimmings and shavings of bovine hides tanned with wet-blue technology was chemically characterized, and its effects in maize (*Zea mays* L.) were evaluated. AA309 contained 13.60% total nitrogen (N), mainly in organic forms (13.40%), and several amino acids, especially lysine, phenylalanine, glycine, aspartate, and isoleucine. AA309 was further analyzed using Fourier Transform Infrared (FT-IR) spectroscopy, which revealed the presence of amide I and amide II bands, indicative of peptide structures. When supplied to maize plants for 15 days at two N dosages (2.1 or 4.2 mg/kg), AA309 induced positive physiological responses, likely because of its content in amino acids functioning as signaling molecules. The low dosage was the most effective in improving leaf (+24%) and root (+98%) dry weight, photosynthetic activity (+70%), and accumulation of N (+80%), proteins (+65–75%) and antioxidants (+60%). Spectroscopic analyses (Solid-state Cross-Polarization Magic Angle Spinning Carbon-13 Nuclear Magnetic Resonance, CP/MAS ^13^C–NMR, and High resolution-magic angle spinning nuclear magnetic resonance, HR-MAS NMR) on plant tissues revealed the increase in proteins, lignin structures and cutin in AA309-treated plants compared to untreated plants. Our results indicate that AA309 could be used as a valuable biostimulant in agriculture.

## 1. Introduction

In the last years, crop productivity has been affected by climate warming, changes in precipitation patterns and a variety of stress conditions, particularly drought and salinity [1]. In addition, the decreased soil fertility and/or high soil degradation caused by unsustainable farming practices that use inorganic fertilizers as the main input of nutrients for plants has been significantly responsible for drops in crop yields [2]. On this account, there is a growing interest in cost-effective, sustainable, and environmentally friendly systems, which may provide high crop productivity and quality [3]. One valuable option is the adoption of biostimulants in agriculture [4,5,6,7]. These products display multifaceted properties, and their use by farmers may restrict the application of chemical fertilizers during tilling practices, thus potentially reducing negative impacts on the environment. Improving the knowledge of how biostimulants influence plant physiology appears critical in order to attain their maximum efficacy for high crop yields.

In 2011, the European Biostimulant Industry Council (EBIC) and the US Biostimulant Coalition (BC) clarified the legislative framework that regulates the biostimulant market and recognized the role of biostimulants in sustainable agriculture. In accordance with the EBIC [8], biostimulants include mixtures of compounds effective in promoting plant growth and development across the crop life cycle through the enhancement of plant metabolism and increased resistance/tolerance to abiotic and biotic stresses, promotion of nutrient and water use efficiency, amelioration of the physic-chemical properties of soils and by prompting complementary soil micro-organism communities’ development and biodiversity.

Biostimulants contain an array of organic and inorganic substances that work synergically to improve plant growth when applied to plants in very low amounts. Based on their origin and chemical composition, they are classified in animal or plant raw materials, seaweed extracts, humic substances, and biomass wastes [4,5,9,10,11]. Given the complexity of mixtures and the broad spectrum of active compounds they contain, biostimulants display a variety of modes of action, which differ from mineral fertilizers [4,5]. Biostimulants’ activity depends on the content in signaling molecules, such as phenols [5,12,13], amino acids [14,15,16], or hormones [17]. However, which biochemical pathways are targeted by biostimulants and how they are regulated is a topic still poorly understood that needs further investigation [18,19,20].

Among biostimulants, protein hydrolysates (PHs) are achieving growing interest [21]. They are mixtures of polypeptides, oligopeptides, and amino acids obtained via chemical and/or enzymatic hydrolysis of proteins [17,19,22,23]. The scope of breaking down the source material into amino acids and small peptides is to produce organic forms of nitrogen (N) that can be available for plant uptake and might act as signaling molecules in developmental processes. Protein hydrolysates may additionally contain hormone like-substances that partly account for their biostimulant activity. For instance, indoleacetic acid (IAA) and triacontanol (TRIA) were previously identified in plant-derived protein hydrolysates by Ertani et al. [17].

The biostimulant properties of PHs are primarily ascribed to their positive effects on N uptake and assimilation, especially via up-regulation of gene expression and activity of enzymes functioning in N metabolism [4,15,19,24]. The PHs-dependent regulation of carbon (C) and N metabolic pathways cross-talk has been reported by Schiavon et al. [15], and a number of studies have described the capacity of protein hydrolysates to enhance the secondary metabolism that produces polyphenols, critical metabolites involved in plant defense responses and tolerance to abiotic and biotic stress [17,25]. Protein hydrolysates can increase the nutritional status of the plant via up-regulation of genes coding for nutrient root membrane transporters and because of their content in certain macronutrients (mainly phosphorus and potassium) and micronutrients, which can be supplied to plants at a steadier rate than mineral fertilizers or synthetic products [14,21,26,27].

Beneficial effects of PHs on plant physiology can also be due to changes they induce on plant-associated microbes’ activity and composition. Amino acids contained in PHs in particular, represent a good source of C and N for rhizosphere microbes, which transform poorly available nutrients to plants in soil into forms that can be more easily taken up, thus leading to enhanced plant nutrient uptake, stronger root systems, and an overall improvement of plant health [21].

To date, most research on biostimulants has focused on products manufactured in liquid form [28], while few studies have assayed the efficacy of solid-formulated products as suitable biostimulants. The benefits offered by solid biostimulants include the slow-release and slow-action of nutrients in the growth medium, which trigger a faster improvement of crop yield. Solid biostimulants may also stimulate the growth of soil microorganisms, especially those that play a central role in plant growth stimulation (i.e., plant growth promoting bacteria, PGPB). In addition, solid biostimulants reduce handling, storage, and transportation costs over other liquid formulations [28].

In this study, a protein-based product in solid form (AA309) was first characterized for its chemical and spectroscopic properties. AA309 was further applied to maize plants in a pot experiment under controlled conditions and tested for its biostimulant activity. Chemical changes in plant composition and effects on growth after treatment with AA309 were assayed. In order to avoid interaction effects due to the soil, maize plants were grown on inert medium.

## 2. Results

### 2.1. Chemical and Spectroscopic Characterization of AA309

The main chemical and physical features of AA309 are shown in Table 1. The amount of total carbon (C) was 400 g/kg, while total nitrogen (N) accounted for 136 g/kg, with a content of inorganic nitrogen in the form of ammonia equal to 2.0 g/kg, and the percent content of organic nitrogen equal to 134.0 g/kg. The value of electric conductivity was 1.08 dS/m. The amount of Zn, Mn, Cu, and K2O was equal to 13.0, 5.5, 5.4, and 130 mg/kg, whereas the content of heavy metals, such as Cr and Cd, was negligible.

Free amino acids detected in AA309 are reported in Table 2. The most abundant amino acids were lysine (0.170 g/kg), aspartate (0.129 g/kg), phenylalanine (0.101 g/kg), glycine (0.098 g/kg), and isoleucine (0.093 g/kg), while cysteine, glutamate, methionine, and arginine were present with intermediate values (0.075, 0.066, 0.060, and 0.053 g/kg, respectively). The content of the remaining amino acids (valine, serine, threonine, alanine, tyrosine) was lower than 0.050 g/kg.

The FTIR spectrum of AA309 showed the characteristic bands of proteins (Figure S1). In particular, the absorption bands at 3069 cm^−1^ and 2926 cm^−1^ were given by NH stretching vibration of NH_2_ and asymmetric stretching vibration of CH_2_ groups. CH_2_ bending and wagging vibration appeared at 1452 cm^−1^ and 1337 cm^−1^, respectively. The bands at 1620 cm^−1^, 1528 cm^−1^ and 1240 cm^−1^ indicated the presence of amide I, amide II and amide III, respectively [29]. Finally, the skeletal stretching vibration appeared at 1078 cm^−1^ and 668 cm^−1^. 

### 2.2. Effect on Leaf and Root Dry Weight

**Figure 1:**
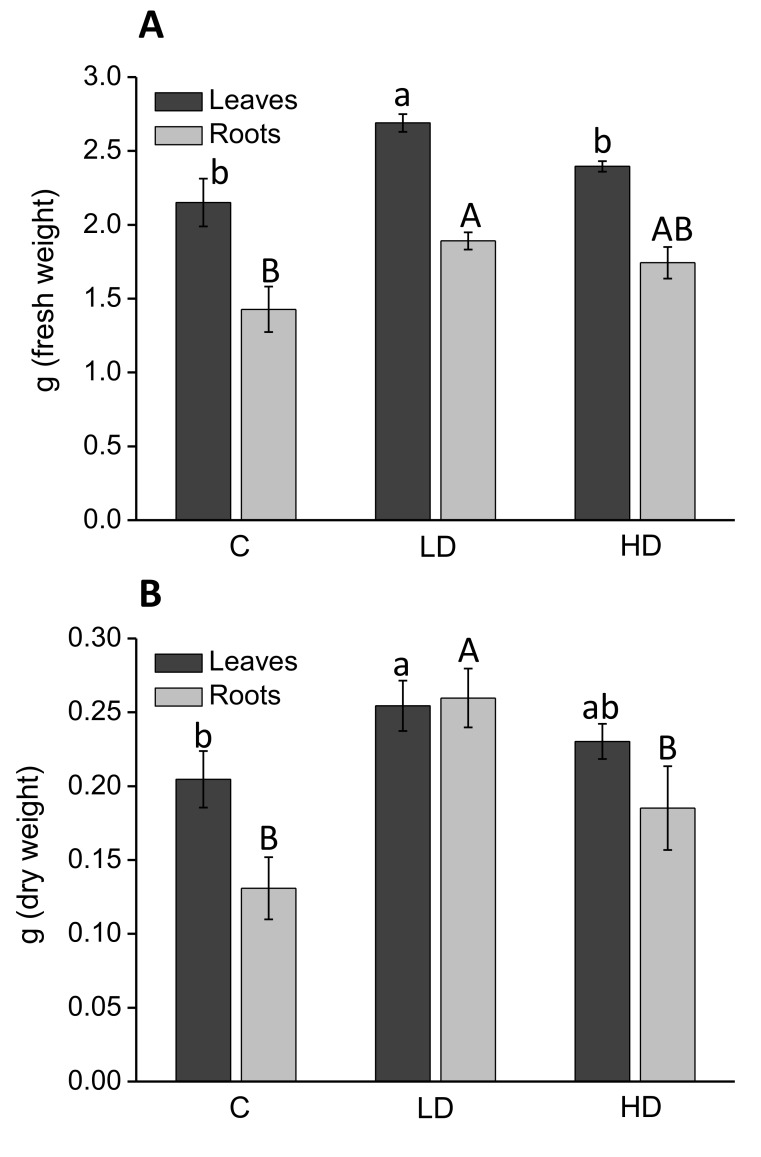
Fresh (**A**) and dry (**B**) leaf and root weight of maize plants grown for 15 days in sand with or without AA309. The weight is referred to the total leaf or root biomass per plant. C = control, LD = Low AA309 dosage (=2.1 mg/kg N); HD = High AA309 dosage (=4.2 mg/kg N). Different letters above bars indicate significant differences between treatments (*p* < 0.05, ±SD, *n* = 10). Lower case letters are referred to leaf comparison, while capital letters are referred to root comparison.

The effect of AA309 on maize plant growth is reported in Figure 1. Both AA309 dosages (corresponding to 2.1 and 4.2 mg/kg N) promoted leaf and root fresh (Figure 1A) and dry weight (Figure 1B). However, AA309 at lower dosage (2.1 mg/kg N) induced a more pronounced increase in biomass production. Specifically, the low AA309 dose enhanced the leaf fresh and dry weight by 20% and 24%, respectively. The root dry weight was also more increased by the low AA309 dose (98%), while the root fresh weight was equally stimulated by the two AA309 dosages (about 20%). In Figure S2, plants are shown as they were in the pots after the experiment).

### 2.3. Effect on SPAD Index, Nitrogen, and Protein Content

**Figure 2:**
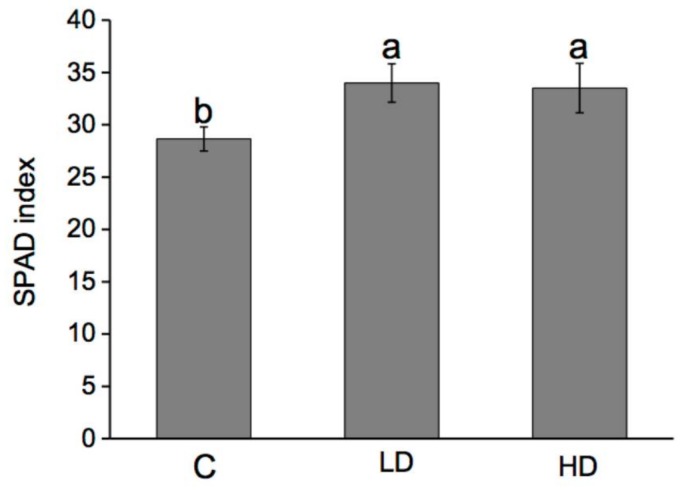
SPAD (Soil Plant Analysis Development) index values measured in maize plants grown for 15 days in sand with or without AA309. C = control, LD = Low AA309 dosage (=2.1 mg/kg N); HD = High AA309 dosage (=4.2 mg/kg N). Different letters above bars indicate significant differences between treatments (*p* < 0.05, ±SD, *n* = 10).

The SPAD index, nitrogen and protein contents were evaluated in order to establish the stimulatory effect of AA309 on maize plants N metabolism. AA309 at low and high dosage enhanced the SPAD index values by 18% and 16%, respectively (Figure 2).

**Figure 3:**
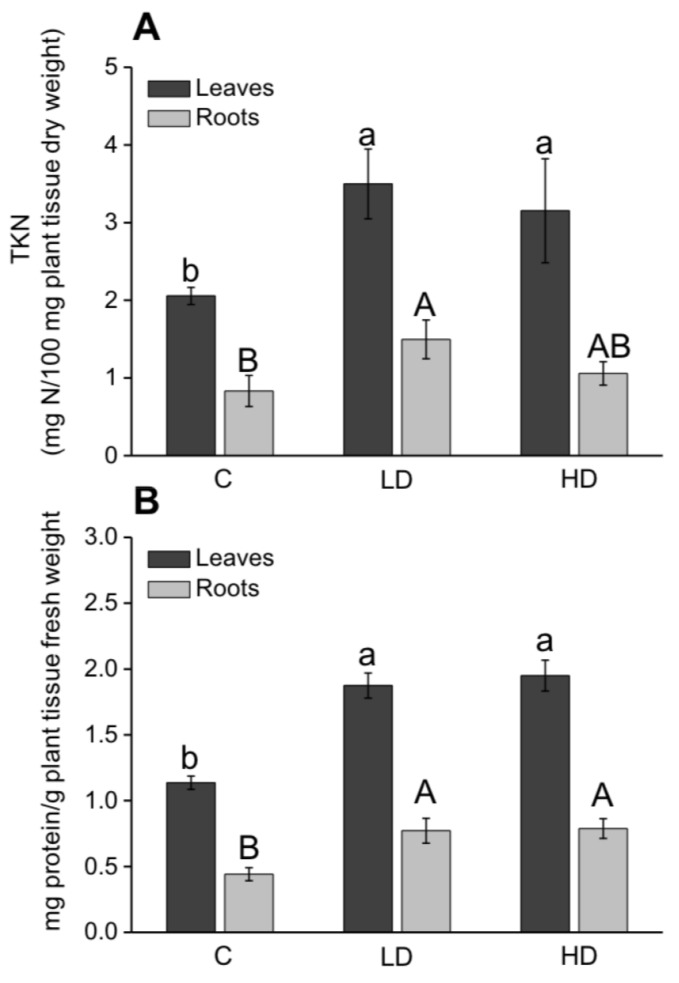
Total Kjeldhal nitrogen (TKN) (**A**) and protein content (**B**) in leaves and roots of maize plants grown for 15 days in sand with or without AA309. C = control, LD = Low AA309 dosage (=2.1 mg/kg N); HD = High AA309 dosage (=4.2 mg/kg N). Different letters above bars indicate significant differences between treatments (*p* < 0.05, ±SD, *n* = 5). Lower case letters are referred to leaf comparison, while capital letters are referred to root comparison.

The amount of total nitrogen in leaves and roots of plants treated with AA309 was greater than in untreated plants (Figure 3A), with maximum values being measured in plants added with a low dosage of AA309 (+70%). The trend observed in roots was the same as in leaves, as total N content increased by 80% and by 27% in plants treated with low and high AA309 dosage, respectively. The protein content was subjected to a stimulation by 65% and 72% in leaves of plants supplied with AA309 at low and high dose, respectively, while in roots the stimulation accounted for 75% and 79%, respectively (Figure 3B).

### 2.4. Analyses of Phenolic Acids

Differential accumulation of phenolic acids—one benzoic acid-derivative (hydroxybenzoic acid) and three cinnamic acid-derivatives (caffeic, *p-*coumaric and ferulic)—was detected in plants treated with AA309 (Table 3). The content of caffeic acid was higher in leaves of plants treated with both AA309 doses compared to untreated plants. In particular, the increment ranged from 60% in plants grown with the low AA309 dose, to 53% in plants treated with the high AA309 dose. Ferulic and hydroxybenzoic acid levels did not change, while *p-*coumaric acid increased (+16%) in leaves of plants supplied with the low AA309 dosage. In roots, a significant accumulation of p-coumaric (+136%), ferulic (13%) and hydroxybenzoic (+76%) acids were observed in plants treated with AA309 at high dosage.

### 2.5. FTIR Spectroscopy on Plant Material

**Figure 4:**
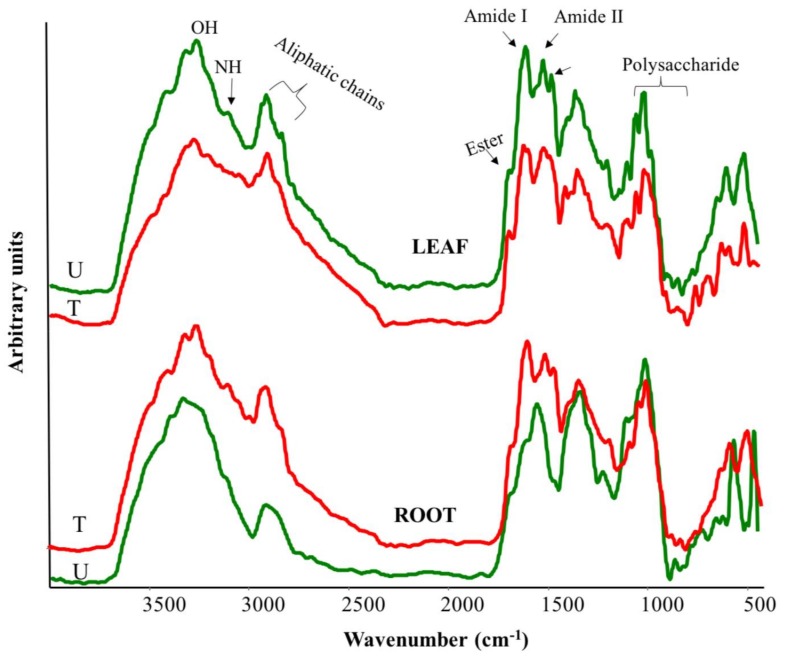
Fourier transform infrared (FTIR) spectra of leaves (top) and roots (bottom) from plant maize untreated and treated with high dosage (=4.2 mg/kg N) of AA309.

Plant tissues are composed of many substances and give complex spectra with many vibrational bands. Due to their complexity, it is not possible to identify each specific compound responsible for individual spectral feature. However, major classes of compounds can be recognized in the samples. In Figure 4, spectra referred to leaves derived from AA309 untreated and treated plants are shown. The samples spectra for plants treated with AA309 at low dosage are not displayed.

In general, in leaf and root spectra, according to Yang et al. [30], the following absorption bands can be distinguished: a region between 3500–3000 cm^−1^, which is dominated by the O–H and N–H stretching vibrations; CH_3_ and CH_2_ stretching vibrations that appear at 3000~2800 cm^−1^; a region between 1800–1200 cm^−1^, which is characterized by C=O stretching vibration (1738 cm^−1^) indicating ester-containing compounds commonly found in membrane lipid (i.e., cutin) and cell wall pectin [31]; amide I (1656 cm^−1^) and amide II (1563 shifted to 1559 cm^−1^) in proteins; at 1513 cm^−1^ vibrations of aromatic ring like lignin derivates; CH_3_ and CH_2_ bending motion at 1460 and 1400 cm^−1^; a region within 1235–1153 cm^−1^, which is due to the C–O stretching in ester and amide III; and in the “fingerprint” region between 1100–1000 cm^−1^, there are several vibrations of groups such as C–H bending or C–O or C–C stretching, which are characteristic of cellulose in the leaves.

Second derivative (2D) enhanced the spectral resolution by amplifying differences in the IR spectra as well as resolving some overlapped absorption peaks (spectra not shown).

The leaves of plants treated with high AA309 dosage showed an evident structural change in the aliphatic region at 2850 and 1453 cm^−1^ in terms of higher intensity compared to leaves derived from the untreated plants. A considerable change also appeared in the protein region due to an enhancement of N–H stretching at 3200 cm^−1^ and 3113 cm^−1^, amide I (1653 cm^−1^) and amide II (1563 cm^−1^) [31,32]. All these modifications were also supported by the content in total protein found (Figure 3). The evidence of a new band at 1516 cm^−1^ suggested the appearance of an aromatic compound as consequence of the treatment. This result was supported by NMR and phenolic analyses. In the carbohydrate region, the bands intensity of ester increased (1737 cm^−1^ and 1248 cm^−1^ and 1157 cm^−1^), and other news bands appeared at 1194 cm^−1^ (C–O–C stretch), 1108 cm^−1^ (C–O, C–C and C–O–C stretch), 937 cm^−1^ (C–O stretch), 840 cm^−1^ (C–O–C skeletal mode) and 708 cm^−1^ (–CH_2_ rocking motion) [31].

In the 2D spectrum of roots of plants treated with high AA309 dosage, a great structural change appeared in the region between 1740–1500 cm^−1^. The bands at 1735 cm^−1^ (esters) and 1644 cm^−1^ (proteins) showed a significant decrease, whereas the band at 1595 cm^−1^, due to aromatic ring vibration, considerably increased.

### 2.6. NMR Spectroscopy

**Figure 5:**
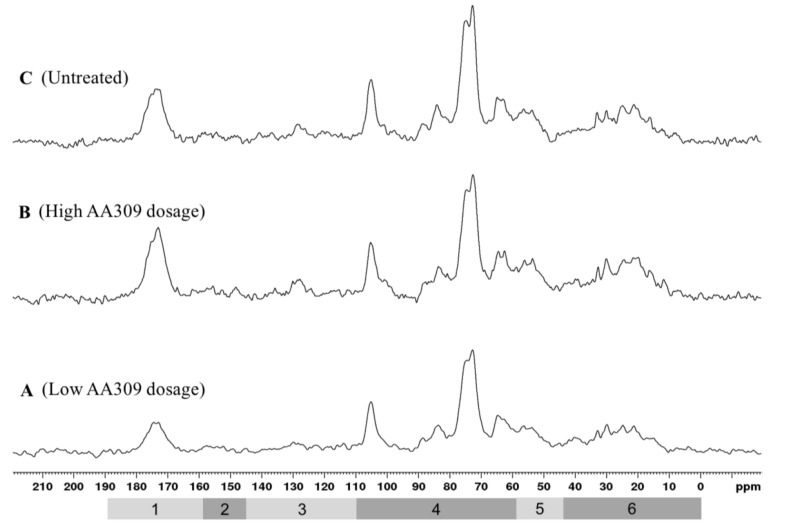
^13^C MAS nuclear magnetic resonance (NMR) spectra of leaves derived from AA309-untreated (control) plants (**A**), plants treated with low AA309 dosage (=2.1 mg/kg N) (**B**) or high AA309 dosage (=4.2 mg/kg N) (**C**) (MASr 25 kHz, 298 K). Region 1: carbonyl-C (esters, amides, aldehydes, carboxylic acids); region 2: phenol-C; region 3: Aryl-C; region 4: *O*-alkyl-C; region 5: methoxyl+alpha-amino-C; region 6: alkyl-C (proteins/lipids, waxes, cutin).

Figure 5 depicts the ^13^C CPMAS NMR spectra of leaf samples. From bottom to top are the spectra of leaves from untreated plants (control) (A), and leaves of plants treated with low (B) and high (C) AA309 dosage. Spectral width can be qualitatively divided into six typical regions, as previously reported [33,34]. For all samples, a qualitative analysis of the spectra suggests the presence of an elevated amount of cellulose and hemicellulose, characterized by the signals at: 105 ppm corresponding to anomeric carbon, 84, 72–73, and 65 ppm due respectively to C-4, C-2/C-3/C-5, and C-6 of pyranoside structures. Samples are rich in aliphatic compounds mainly attributed to proteins, cutin, cell walls and waxes. Actually, broad signals in region 6 may be attributed to long methylene chains in cutin.

In order to better evaluate the effects on leaf chemical compositions induced by the supply of AA309 to plants, ^13^C spectra were normalized with respect to carbohydrates content using the peak at 73 ppm as a normalizing signal (data not shown). Cutin, hemicellulose, proteins, extractive lipids, and, to a minor extent, benzoic and cinnamic acids derivatives may contribute to the increased intensity of region 1. The increase observed in region 5 may be attributed to carbon attached to nitrogen in amino acid moieties, confirming a greater intake of N by AA309 treated plants compared to the untreated controls. Finally, region 6 (Aliphatic-C) was positively affected by the treatment, showing both an increase in intensity and line broadening, which may account for an elongation of –CH_2_– chains derived from various compounds, primarily proteins, but also lipids, plant waxes, and polyesters. ^13^C CPMAS NMR spectra of roots showed only slight variation in the chemical composition of between AA309 untreated and treated plants.

**Figure 6:**
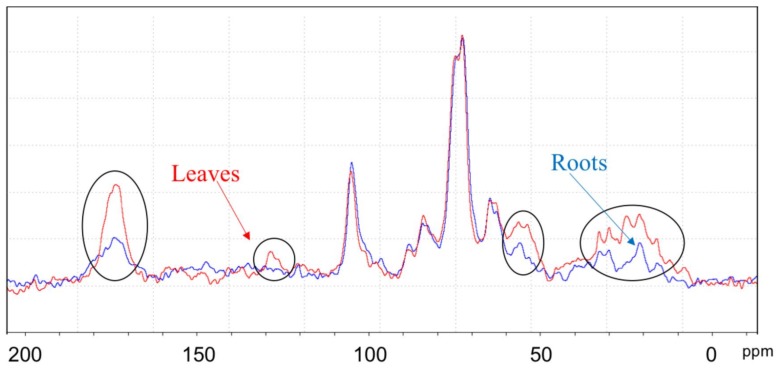
^13^C MAS NMR spectra of leaves (red) and roots (blue) derived from plants treated with high AA309 (=4.2 mg/kg N) dosage (MASr 25 kHz, 298 K). Ellipsis highlight the resonances that are increased in leaves with respect to roots; spectra are normalized on carbohydrate content.

**Figure 7:**
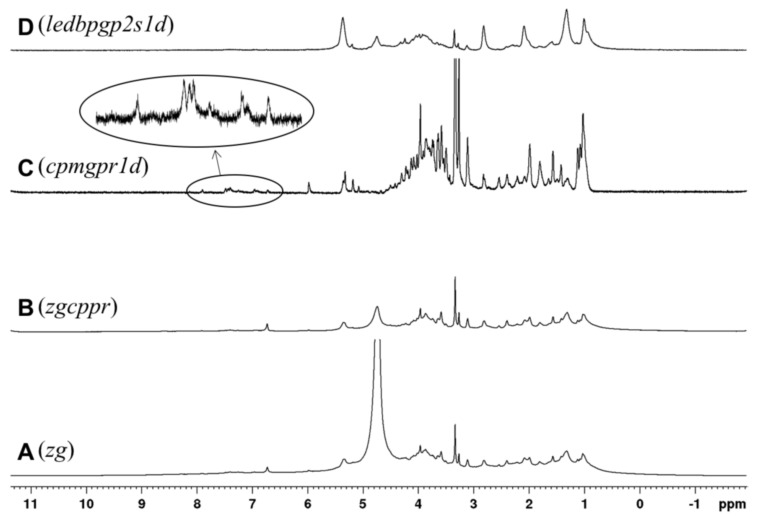
^1^H HRMAS NMR spectra of leaves derived from plants treated with high AA309 (=4.2 mg/kg N) dosage in D_2_O (MASr 12 kHz, 298 K), from bottom to top: (**A**) zg; (**B**) zgcppr; (**C**) cpmgpr1d; (**D**) ledbpgp2s1d.

**Figure 8:**
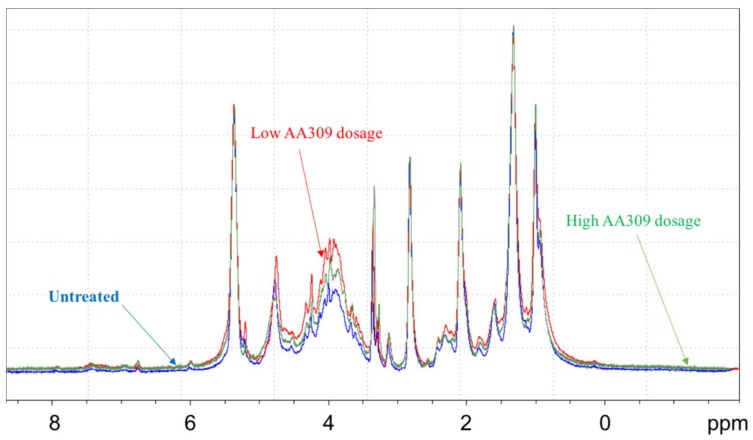
^1^H HRMAS NMR spectra of leaves in D_2_O (MASr 12 kHz, 298 K) acquired with the Bruker sequence ledbpgp2s1d. Different colours are referred to different treatments to which plants were subjected: AA309 untreated (control, blue), low AA309 dosage (=2.1 mg/kg N) (red), high AA309 dosage (=4.2 mg/kg N) (green).

Comparing the spectra of leaves and roots from AA309 treated plants (Figure 6), its leaves appeared to be more enriched in N-containing species, particularly polypeptides and proteins, as suggested by intense resonances in region 1 and region 5. These findings are consistent with analytical data concerning N and protein concentrations in leaves and roots (Figure 3A,B). Leaves were also characterized by a higher content in aromatic species, probably lignin structures, as supported by the broad resonance in region 3, as well as aliphatic species, especially proteins and esters-like cutin, lipids, and waxes of cell walls. In order to explore the effect of the AA309 treatment on the chemical composition of the more hydrophilic species, leaf and root samples were investigated by means of HR MAS ^1^H NMR after addition of D_2_O. As previously reported, this technique elucidates the presence of compounds that are at the interface between the solid and liquid, or that are soluble in the applied solvent. Figure 6 shows the ^1^H HR MAS NMR spectra of the leaf samples of plants treated with AA309 at low dosage. The application of different pulse sequences, such as cpmgpr1d and ledbpgp2s1d, helps to separate the contributions on the whole spectrum due to small molecules with high mobility (phenols, amino acids, small peptides, simple carbohydrates, etc.) and to high molecular weight structures (polypeptides, proteins, long chain lipids, and waxes). The cpmg sequence highlighted the presence of low molecular weight aromatic species (spectrum C—Figure 7), probably phenols, in small amounts. This indicated that total aromatic compounds were not major constituents of this fraction, which may consist of soluble hemicelluloses for the main. The comparison between untreated and treated leaves (Figure 7 and Figure 8) evidenced an increment in the region 3.7–4.2 ppm, attributed to an increase in water soluble amino acids and small peptides.

## 3. Discussion

In this study, a solid protein hydrolysate of animal origin, AA309, was applied to maize plants to evaluate its biostimulant properties. AA309 contained several amino acids that can likely be absorbed by plants via root amino acids plasma membrane transporters and further translocated throughout the plant [14,35,36]. The essential amino acid lysine, which was the most abundant in AA309, deserves a special mention because it is generally limiting in cereal crops, therefore causing severe nutritional and economical consequences worldwide. Lysine/histidine transporters (LHTs) may possibly mediate lysine transport into maize root cells [37].

Overall, amino acids furnished by AA309 can act as signaling molecules by eliciting transduction pathways that lead to remarkable physiological and biochemical changes in plants [38]. Also, synergically with low-molecular size peptides, they can promote the biosynthesis of endogenous phytohormones that control plant development [39].

Our results indicate that AA309 was able to stimulate plant growth consistently with the promotion of photosynthetic efficiency. Higher photosynthetic rates in plants treated with biostimulants have been previously ascribed to increased activity of the RUBISCO enzyme [18], and/or to their content in molecules with hormone-like activities [40]. Stimulation of plant growth by AA309 could be additionally due to higher accumulation of proteins, as a result of enhanced nitrogen intake and assimilation and/or absorption of amino acids. In support of this, the qualitative analysis of the ^13^C NMR spectra indicated the presence of appreciable amounts of carbonyl, aryl and aliphatic carbons and the increase in peptides and protein content in plants treated with AA309, while the IR spectra highlighted the abundance of amide I (1653 cm^−1^) and amide II (1563 cm^−1^) functional groups in leaves. These findings are in agreement with earlier studies, in which protein hydrolysates-based biostimulants up-regulated the expression of genes involved in N transport and assimilation and prompted the accumulation and synthesis of N-related compounds in plants [15,26,27,41]. It can be hypothesized that amino acids contained in protein hydrolysates, including AA309, provide carbon skeletons to be converted into precursors or intermediates of the tricarboxylic acid cycle, therefore contributing to the respiratory metabolism and ATP production for energy-requiring processes, including nitrogen transport [42,43].

Beside the positive effects on plant growth, photosynthetic efficiency, and accumulation of N compounds, AA309 increased the accumulation of phenolic acids, thus providing further evidence to the current literature that polyphenols biosynthesis is a primary target of protein hydrolysates-based biostimulants in plants [12,18,26,44]. Phenylalanine (Phe), the amino acid precursor of polyphenols in plants, was abundantly present in AA309 as compared to other amino acids and could be responsible for this increase. Accumulation of phenol metabolites within a safe range is a positive effect exerted by AA309 in maize plants, as these compounds are crucial for plants growth and reproduction, synthesis of lignin and pigments, and play a potential protective role against invading organisms and different kinds of oxidative stress [45].

## 4. Material and Methods

### 4.1. Chemical Characterization of the Protein Hydrolysate

AA309 is a solid protein hydrolysate obtained through thermobaric hydrolysis applied on trimmings and shavings of bovine hides previously tanned with wet-blue technology. The hydrolysis process was made in spherical rotating autoclaves by high-pressure steam and followed by a low temperature dehydration system. The determination of elemental components and physical properties of AA309 (Table 1) was performed according to the standard analytical procedures. Moisture was determined by weight loss at 105 °C; ash by residue on ignition at 550 °C; organic matter (OM) by loss on ignition (OM = dry matter-ash); pH in water (3/50, *w*/*v*); total nitrogen (TKN) via Kjeldahl method; and ammonium nitrogen (NH_4_^+^-N) by extraction with diluted HCl and steam distillation with magnesium oxide. Free amino acids (FAA) were extracted using 0.1 M HCl for 1 h and determined with RP-HPLC after derivatization with FMOC (Table 2).

#### FTIR Analysis of AA309

The Fourier transform infrared (FTIR) spectrum of AA309 was recorded with a Bruker TENSOR series FT-IR Spectrophotometer (Bruker, Ettlingen, Germany) equipped with an apparatus for diffuse reflectance (DRIFT) (Spectra-Tech. Inc., Stamford, CT, USA). The spectrum was collected as Kubelka-Munk units using KBr (Aldrich Chemical Co., Milwaukee, WI, USA) as a background reference. The analysis was performed on powdered sample. The sample was mixed with KBr (5:100 *w*/*w*) in an agate mortar. The spectrum was collected between 4000–400 cm^−1^ and averaged over 100 scans (resolution 4 cm^−1^). The spectral data were processed with Grams/386 spectroscopic software (Galactic Industries, Salem, NH, USA). Second derivative was applied by using Grams/386 spectroscopic software (Galactic Industries, Salem, NH, USA) in order to enhance the separation of overlapping peaks.

### 4.2. Plant Material

#### 4.2.1. Growth Conditions

Maize seeds (*Zea mays* L., cv. P1921) provided by Pioneer were soaked for one night in distilled running water and let to germinate for 60 h in the dark at 25 °C on filter paper soaked with 1 mmol/L CaSO_4_ [45]. Subsequently, germinated seeds were transplanted in pots containing sand inside a climatic chamber with a 14 h light/10 h dark cycle, air temperature of 27/21 °C, relative humidity of 70/85% and photosynthetically active radiation (PAR) of 280 mol m^−2^ s^−1^.

Pots containing sand (700 g each) and AA309 were prepared 5 days before the beginning of the experiment, and distilled water (50 mL) was added to each pot in order to allow the slow release of nitrogenous substances from AA309 Before being distributed in the pots, the amount of AA309, was divided into 3 subunits for each concentration and then gradually applied every 5 cm of sand.

The AA309 was supplied to plants at two different dosages of nitrogen (N) (2.1 and 4.2 mg/kg N, respectively). To exclude a fertilizer effect of AA309 on plants, the N furnished by AA309 was subtracted from the Hoagland modified nutrient solution that was furnished to plants during the entire growth period, considering that plants were irrigated with 100 mL every 2 days. The nutrient solution had the following composition (μmol/L): Ca(NO_3_)_2_ (200), KNO_3_ (200), MgSO_4_ (200), KH_2_PO_4_ (40), FeNaEDTA (10), H_3_BO_3_ (4.6), MnCl_2_ (0.9), ZnCl_2_ (0.09), CuCl_2_ (0.036), NaMoO_4_ (0.01). Plants grown in the absence of AA309 served as controls. For each treatment, ten pots were prepared, with one plant in each.

After 15 days from the transplant, plants were randomly harvested, and roots were carefully washed to remove sand particles and dried with blotting paper. A sub-sample of the plant material was immediately frozen with liquid nitrogen and kept at −80 °C for physiological analyses. For fresh and dry weight measurement, ten plants per individual treatment were used. After measuring the fresh weight, root and leaf samples of individual plants were placed in a drying oven for 2 d at 70 °C, allowed to cool for 2 h inside a closed bell jar and weighed separately.

With respect to elemental and spectroscopic analyses, leaf and root dry samples were lyophilized and ground in a cyclone grinder to pass a 20 mesh screen.

#### 4.2.2. SPAD Index, Nitrogen, Protein, and Phenolics

The quantification of relative chlorophyll concentration was performed through a non-destructive method that uses light transmission through a leaf, at two wavelengths, to determine the greenness and thickness of leaves. Transmission in the infrared range provides a measurement related to leaf thickness, and a wavelength in the red light range is used to determine greenness. The ratio of the transmission of the two wavelengths provides a chlorophyll content index that is referred to as a SPAD index [46]. A soil plant analysis development (SPAD) chlorophyll meter (SPAD-502 model, Minolta Camera Co., Ltd., Osaka, Japan) was used to measure the SPAD index on the last expanded leaf of maize plants. The determination was carried out on five measurements per leaf from 10 plants per experimental condition.

For the extraction of soluble proteins, frozen foliar and root tissues (500 mg) were ground in liquid nitrogen, vortexed with 5 mL extraction buffer (100 mmol/L Tris HCl pH 7.5, 1 mmol/L Na_2_EDTA, 5 mmol/L DTT), and centrifuged at 14,000 *g* for 15 min. The supernatants were mixed with 10% (*w*/*v*) trichloroacetic acid and centrifuged. The pellets obtained were re-suspended in 0.1 N NaOH. The protein concentration on five replicates was analyzed using a UV/VIS spectrophotometer (Lambda 1, Perkin-Elmer, Monza, Italy) at λ = 595 nm according to Bradford [47] and expressed as mg protein per g fresh weight.

Standard Kjeldahl method was performed for determining total N in tissues. Five replicates for each treatment were used, and total N was expressed as weight dry percent (% *w*/*w*).

The amount of phenolic acids was determined in leaves and roots of three plants as described by Ertani et al. [43].

#### 4.2.3. FITR and NMR Spectroscopies

FTIR spectra were determined on lyophilized tissues according to the method reported in the paragraph 2.1.1. Nuclear magnetic resonance (NMR) spectra were recorded with a Bruker FT-NMR AVANCE III HD 600 MHz spectrometer at 298 K; the Larmor frequencies of proton and carbon were 600.13 MHz and 150.90 MHz for ^1^H and ^13^C, respectively. Each sample was acquired in both solid state and in semi-solid state, using respectively a 2.5 mm double resonance H-X CP-MAS probe and 4 mm ^1^H/^13^C/^31^P HR MAS probe equipped with ^2^H lock and Z gradients. 

##### Solid-State MAS NMR

The ^1^H and ^13^C spectra were externally referenced using glycine. All samples were finely crushed in an agate mortar and introduced into a 12 L MAS zirconium rotor (2.5 mm OD) and finally transferred into the MAS probe. All samples were spun at the magic angle with a spinning rate of 25 kHz during acquisition. At this spinning rate first order spinning side bands (SAA309^1^) occur outside the spectral region of interest. Proton spectra were run using the typical Bruker *onepulse* sequence (4 scans). Standard cross-polarization MAS pulse sequence (*cp* in Bruker library) with high-power proton decoupling during acquisition was applied to obtain ^13^C spectra. Longitudinal delay (T1) was predicted by standard *t1ir* Bruker sequence, for all samples T1 was below 1s, hence relaxation delay (D1) was typically set to 5 s. For ^13^C the number of scans ranged within 128-2k. Contact time (P15) range was between 0.5–6 ms.

##### Semi-Solid-State HR MAS NMR

Ten mg of each lyophilized sample were introduced into HR-MAS zirconium rotor (4 mm OD) equipped with an insert to reduce internal volume (12 L), and 5 L of D_2_O were added to completely wet the sample. The rotor was then transferred into the MAS probe and spun at the magic angle at 12 kHz. An internal lock on the deuterium of D_2_O was used for all spectra. The chemical shifts were referred to TMS. 1D NMR data were acquired using standard Bruker pulse sequences: *zg* (1D sequence), *zgcppr* (1D sequence with pre-saturation and using composite pulse for selection), *ledbpgp2s1d* (bipolar longitudinal eddy current delay pulse sequence-BPPLED), and *cpmgpr1d* (Carr-Purcell-Meiboom-Gill-CPMG). Second derivative homo- and hetero-correlation experiments were acquired using typical Bruker pulse sequences *cosygpppqf* (2D homonuclear shift correlation using gradient pulses for selection and purge pulses before relaxation delay), *mlevphpr* (phase sensitive homonuclear Hartman-Hahn transfer using MLEV17 sequence for mixing, two power levels for excitation, and spinlock), and *hsqcedetgpsp* (phase sensitive 2D ^1^H, ^13^C HSQC performed via double INEPT transfer using Echo/Antiecho-TPPI gradient selection, decoupling during acquisition, performed using TRIM pulses in INEPT transfer with multiplicity editing during the selection step).

### 4.3. Statistical Analysis

For all determinations, the analysis of variance (ANOVA) was performed using the SPSS software version 18.0 (SPSS, Chicago, IL, USA), and was followed by pair-wise post-hoc analyses (Student-Newman-Keuls test) [48] to determine which means differed significantly at *p* < 0.05 (*±*SD).

## 5. Conclusions

The present work shows that the solid product AA309 stimulated different aspects of plant metabolism, such a plant growth, accumulation of N, proteins, and antioxidants (phenolics), perhaps by virtue of its content in small peptides and amino acids that can be absorbed by roots and further act as signaling molecules. Therefore, AA309 displays potential as biostimulant for use in agricultural practices for the successful promotion of plant yields. 

## Figures and Tables

**Table 1:** Chemical properties of AA309.

Property	°	Unit
pH	4	
Electric conductivity	1.08	dS/m
Ash	81.0	g/kg
Organic N	134.0	g/kg
NH_4_^+^	2.0	g/kg
Total N (TN)	136.0	g/kg
Total C (TC)	400.0	g/kg
Total SO_3_	47.0	g/kg
Na_2_O	10.0	g/kg
CaO	6.7	g/kg
Cr	2.6	g/kg
K_2_O	130	mg/kg
Cu	5.40	mg/kg
Cd	<1	mg/kg
Pb	<5	mg/kg
Ni	2.00	mg/kg
Zn	13.00	mg/kg
Mn	5.50	mg/kg

**Table 2:** Amino acid concentrations in AA309.

Amino Acid	g/kg
Aspartate	0.129
Glutamate	0.066
Serine	0.027
Glycine	0.099
Threonine	0.026
Arginine	0.053
Alanine	0.038
Tyrosine	0.033
Cysteine	0.075
Valine	0.048
Methionine	0.060
Phenylalanine	0.101
Isoleucine	0.093
Leucine	0.037
Lysine	0.170

**Table 3:** Content of the most abundant phenolic compounds detected in leaves and roots of maize plants grown for 15 days in sand in the presence or not of AA309. C = untreated plants; LD = Low AA309 dosage; HD = High AA309 dosage.

Caffeic				*p-*Coumaric			Ferulic			Hydroxybenzoic		
Treatment	µg/g dw											
			*Leaves*									
C	26.91	±2.22 ^c^*	95.70		±5.23 ^a^	32.39		±8.22 ^a^	97.05			±21.54 ^a^
LD	43.05	±1.54 ^b^	111.03		±13.52 ^a^	21.34		±8.56 ^a^	86.55			±19.02 ^a^
HD	67.96	±5.23 ^a^	75.19		±11.23 ^a^	20.80		±9.21 ^a^	95.67			±16.63 ^a^
			*Roots*									
C	19.01	±3.12 ^a^	21.83		±5.23 ^b^	8.79		±0.56 ^a^	21.66		±2.77 ^b^	
LD	6.62	±0.98 ^c^	67.22		±12.88 ^a^	3.68		±0.12 ^b^	81.57		±12.03 ^a^	
HD	14.89	±1.05 ^b^	95.09		±15.26 ^a^	9.97		±0.65 ^a^	38.07		±8.28 ^b^	

* Different letters along columns indicate significant differences (*p* < 0.05, ±SD) between treatments.

## Supplementary Materials

Figure S1. FTIR spectrum of AA309; Figure S2. Maize plants grown for 15 days in sand with or without AA309. C = control, LD = Low AA309 dosage (=2.1 mg/kg N); HD = High AA309 dosage (=4.2 mg/kg N).
