# Supplementary material for: Spectroscopic-Chemical Fingerprint and Biostimulant Activity of a Protein-Based Product in Solid Form
Source: Molecules. 2018 Apr 27;23(5):1031. doi: 10.3390/molecules23051031 (PMC6102567; doi:10.3390/molecules23051031)
Supplement: Supplementary file 1 [file molecules-23-01031-s001.pdf]

Supplementary material

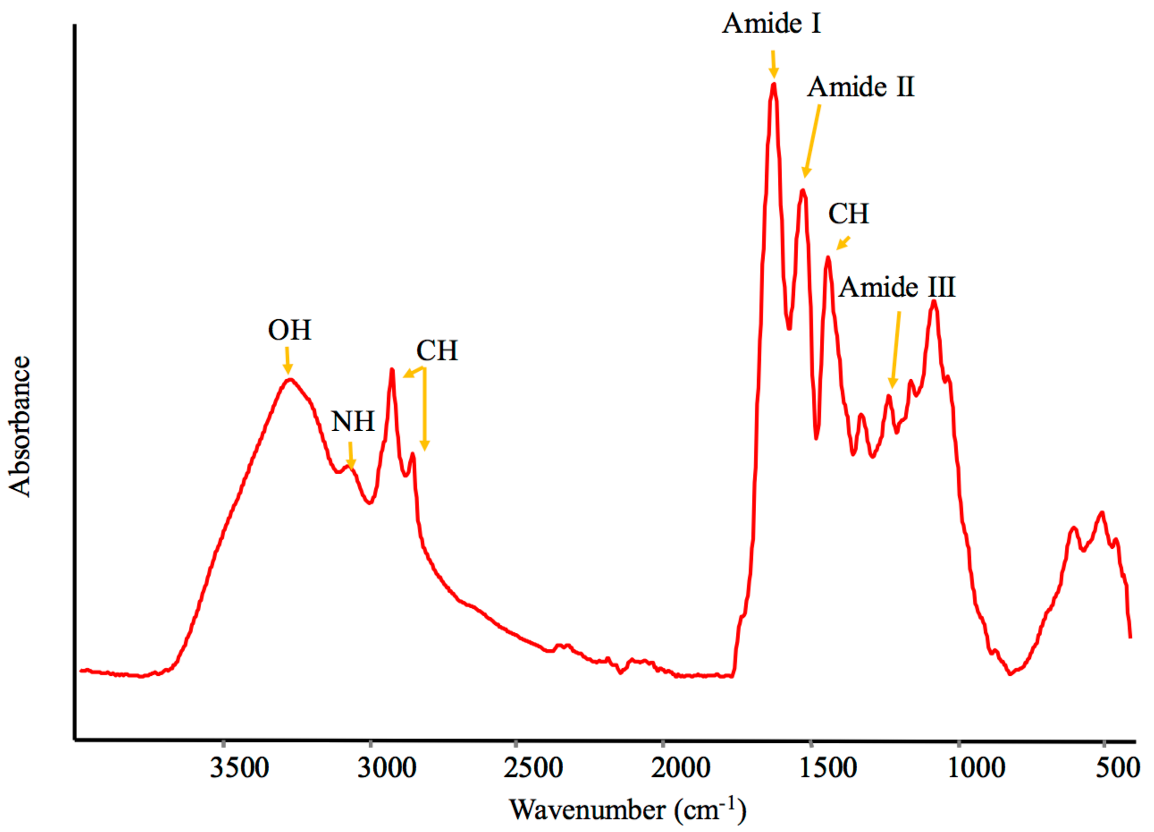

Figure 1S. FT-IR spectrum of AA309.

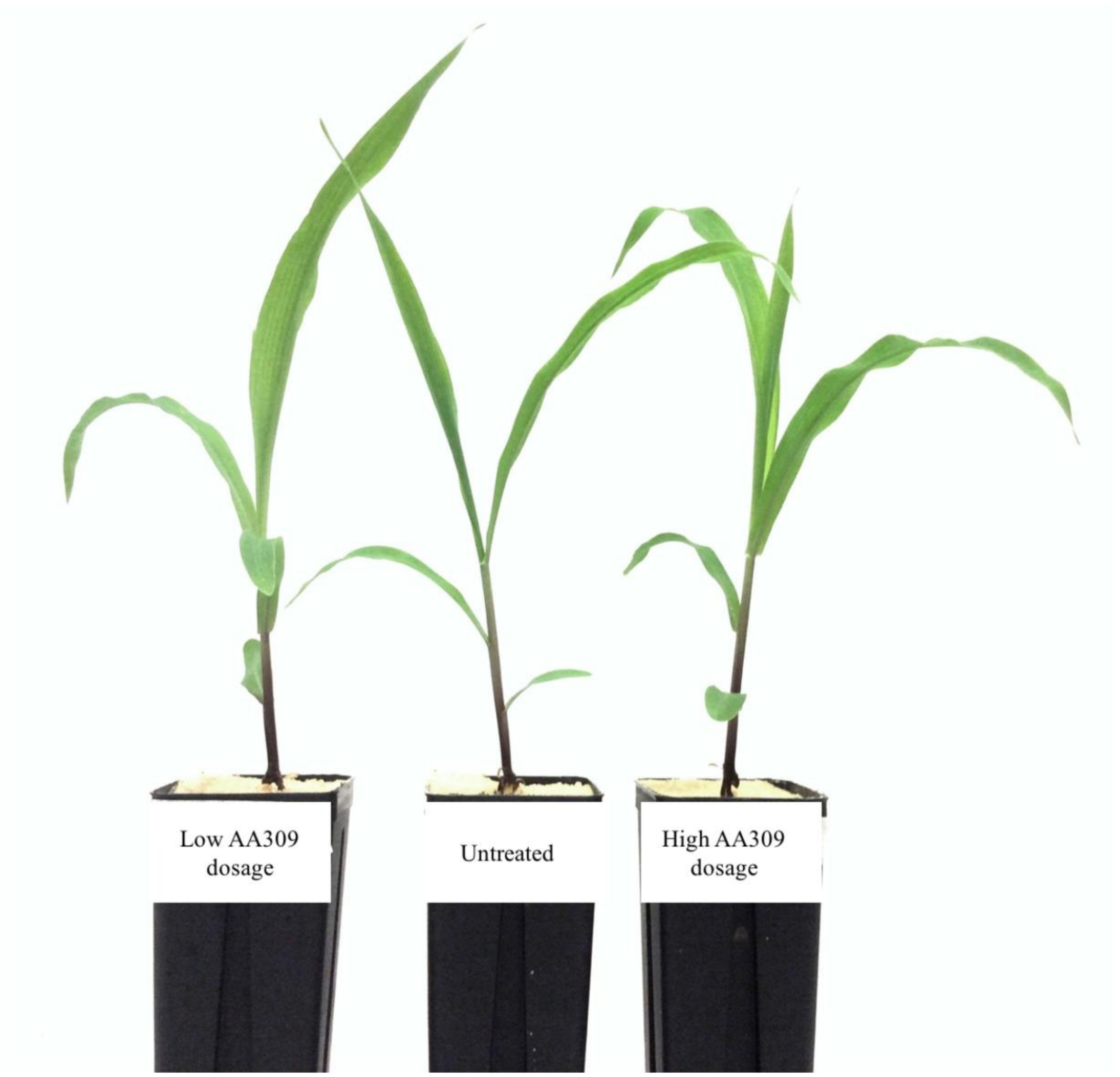

Figure 2S. Maize plants grown for 15 days in sand with or without AA309. C=control, LD= Low AA309 dosage (= 2.1 mg/kg N); HD= High AA309 dosage (= 4.2 mg/kg N).
